# Spasticity Measurement Based on Tonic Stretch Reflex Threshold in Children with Cerebral Palsy Using the PediAnklebot

**DOI:** 10.3389/fnhum.2017.00277

**Published:** 2017-05-29

**Authors:** Marco Germanotta, Juri Taborri, Stefano Rossi, Flaminia Frascarelli, Eduardo Palermo, Paolo Cappa, Enrico Castelli, Maurizio Petrarca

**Affiliations:** ^1^Don Carlo Gnocchi Onlus FoundationMilan, Italy; ^2^Department of Mechanical and Aerospace Engineering, “Sapienza” University of RomeRome, Italy; ^3^Industrial Engineering, Department of Economics and Management, University of TusciaViterbo, Italy; ^4^Neurorehabilitation Units, Movement Analysis and Robotics Laboratory, IRCCS Bambino Gesù Children's HospitalRome, Italy

**Keywords:** stretch reflex, spasticity, cerebral palsy, pediAnklebot, modified ashworth scale, tonic stretch reflex threshold

## Abstract

Nowadays, objective measures are becoming prominent in spasticity assessment, to overcome limitations of clinical scales. Among others, Tonic Stretch Reflex Threshold (TSRT) showed promising results. Previous studies demonstrated the validity and reliability of TSRT in spasticity assessment at elbow and ankle joints in adults. Purposes of the present study were to assess: (i) the feasibility of measuring TSRT to evaluate spasticity at the ankle joint in children with Cerebral Palsy (CP), and (ii) the correlation between objective measures and clinical scores. A mechatronic device, the pediAnklebot, was used to impose 50 passive stretches to the ankle of 10 children with CP and 3 healthy children, to elicit muscles response at 5 different velocities. Surface electromyography, angles, and angular velocities were recorded to compute dynamic stretch reflex threshold; TSRT was computed with a linear regression through angles and angular velocities. TSRTs for the most affected side of children with CP resulted into the biomechanical range (95.7 ± 12.9° and 86.7 ± 17.4° for Medial and Lateral Gastrocnemius, and 75.9 ± 12.5° for Tibialis Anterior). In three patients, the stretch reflex was not elicited in the less affected side. TSRTs were outside the biomechanical range in healthy children. However, no correlation was found between clinical scores and TSRT values. Here, we demonstrated the capability of TSRT to discriminate between spastic and non-spastic muscles, while no significant outcomes were found for the dorsiflexor muscle.

## Introduction

Cerebral Palsy (CP) groups a number of non-progressive, yet changing, neurological disorders resulting from brain lesions arising in the early stages of the development (Bax and Goldstein, 2005). It is characterized by different forms of hypertonia with symptoms defined by three main descriptive terms: spasticity, dystonia, and rigidity (Sanger et al., 2003; Ivanhoe and Reistetter, 2004; Sheean and McGuire, 2009). The spasticity, in turn, is commonly defined as a hyperexcitability of the stretch reflex responses, which are velocity-dependent. More specifically, spasticity can be identified as an increasing resistance with the increase of the joint passive rotation or as a rapid rise of the resistance above a threshold speed or joint angle value (Lance, 1981; Sanger et al., 2003; Eek and Himmelmann, 2016).

In clinical setting, spasticity is usually assessed through the Modified Ashworth Scale (MAS) (Bohannon and Smith, 1987). However, its validity and reliability as a measure of spasticity has been questioned (Pandyan et al., 1999; Fleuren et al., 2009). In addition, no significant correlation was found between MAS and measurements of neural and muscular components of joint static and dynamic stiffness (Alibiglou et al., 2008). The main limit of the spasticity assessment using MAS is the impossibility to distinguish between neural and non-neural components. In fact, the mechanical resistance perceived by the examiner is not only addressable to the active muscle response against induced elongation, but also to the elastic and viscous characteristics of muscles, tendons, soft tissues and ligaments. MAS does not take into account the speed dependence of the stretches in induced passive movements, a potential effective parameter for assessing the neural component of spasticity (Phadke et al., 2015). Consequently, MAS can be effectively used as an index of the resistance to passive movements rather than spasticity (Pandyan et al., 1999). Among the clinical scales for spasticity assessment, the Tardieu Scale represents a potential alternative to the MAS scale, as it also considers velocity-dependent characteristic of stretch reflex (Tardieu et al., 1954; Patrick and Ada, 2006). However, it is affected by operator's skills in administration, i.e., the ability to manually impose angular velocity, and by the difficulty to induce fast velocities in children (Mackey et al., 2004). Thus, its reliability is still questioned (Haugh et al., 2006).

Even though clinical scales still represent an integral part of neurological and clinical examination, it is widely acknowledged that instrumented approaches are desirable, to overcome the intrinsic limit of the clinical approach based on “feel the resistance.” Several objective approaches based on the analysis of the exerted force and the muscle activity were proposed to assess spasticity (Schmit et al., 1999; Calota et al., 2008; Poon and Hui-Chan, 2009; Vlugt et al., 2010; Wu et al., 2010). In some studies, the spastic limb was manually moved by an expert operator, while the angular displacement and velocity were measured by sensors, such as electrogoniometers (McGibbon et al., 2013) or inertial measurement units (Bar-On et al., 2013), placed on the subjects. In others, the targeted limb was moved around the anatomical joint by mechatronic devices (Vlugt et al., 2010) to standardize and automate the imposed input and improve the test-retest reliability. With this approach, joint displacements, exerted forces, and muscle activities are measured via sensor systems embedded in mechatronic devices (Kim et al., 2005; Bar-On et al., 2013). Such quantitative and robust measurements could become a valuable tool to reliably monitor patients' progress and assess efficacy of current clinical approaches in spasticity management.

Among the proposed metrics based on sensor outputs, the Tonic Stretch Reflex Threshold (TSRT) index seems to be the more promising approach (Calota and Levin, 2009; Mullick et al., 2012), as it more accurately reflects the Lance's definition of spasticity (Lance, 1981) than other clinical tests (Phadke et al., 2015). In particular, TSRT is estimated by a linear regression model on the Dynamic Stretch Reflex Threshold (DSRT) values, corresponding to the points on a phase diagram (joint velocity vs. joint angle) at which motoneurons and relative muscles begin to be recruited (Calota and Levin, 2009). Levin and Feldman (1994) and Mullick et al. (2012) demonstrated the correlation between TSRT and the degree of spasticity at the elbow joint in adult population. Levin and colleagues also verified that TSRT angle lies within the biomechanical range of motion (ROM) of the joint in subjects with spasticity, while it is outside those limits in healthy subjects. The TSRT value and the slope μ of the regression line were effectively used to evaluate the hyperton at the elbow joint, both in patients with stroke (Calota and Levin, 2009) and CP (Jobin and Levin, 2000), showing moderate to good values of reliability in both cases. TSRT and μ values were also used to discriminate between neurological deficits of muscle tone at the elbow joint in patients with stroke and Parkinson's disease (Mullick et al., 2012). Recently, Blanchette et al. (2015) extended the TSRT approach to the ankle joint as it is one of the most affected joint in patients with spasticity and it significantly affects gait (Wu et al., 2011). Blanchette and colleagues found a high inter-evaluator reliability (ICC = 0.85) for plantarflexor spasticity in adult post stroke patients.

As regards children with CP, ankle spasticity was extensively studied using several approaches (Poon and Hui-Chan, 2009; Vlugt et al., 2010; Bar-On et al., 2013; Schless et al., 2015) based on the manual imposition of passive displacements of the foot and the analysis of the SEMG signal. Even though some researchers (Engsberg et al., 2007; Sloot et al., 2015) proposed the evaluation of spasticity in CP population using a mechatronic device, no studies, to the authors' knowledge, examined the spasticity of plantarflexor and dorsiflexor muscles in pediatric population by means of a mechatronic device, applying the TSRT approach.

Hence, the aim of the present work is twofold. Firstly, we seek to evaluate if TSRT approach can be adopted for the evaluation of the ankle spasticity in children with CP, considering both plantarflexor and dorsiflexor muscles. Secondly, we want to assess if there is a correlation between MAS and quantitative measures obtained from the TSRT approach. For both aims, we used the pediAnklebot (InMotion Technologies, Watertown, MA, USA) (Krebs et al., 2011; Michmizos et al., 2015), a robotic device, to impose passive stretches to the ankle joint measuring both rotation and angular velocity.

## Materials and methods

### Participants

A cohort of ten patients was recruited (6.4 ± 1.8 years, range: 5–9), eight children with hemiplegia (HC) and two with diplegia (DC). Inclusion criteria for patients were: (i) congenital or acquired hemiplegia or diplegia, excluding those with acute events in the last 6 months; (ii) age between 5 and 10 years; (iii) no cognitive nor visual impairments; (iv) no botulinum toxin injection prior 6 months; and, (v) no history of functional surgery. A cohort of three age-matched (Pacilli et al., 2014) typically developing children (TDC) (6.3 ± 0.6 years, range 6–7) was enrolled. TDC met the following inclusion criteria: absence of neurological or musculoskeletal disorders, long term medications, bone lesions or joint pathologies of the lower limbs in the year prior to the study. TDC were enrolled to verify the robustness of the algorithm to false positives, i.e., the erroneous individuation of spasticity level in healthy subjects. More details of the two groups and the MAS assessment of the Gastrocnemius are reported in the Table 1. All subjects were naive to the robotic device and the task. Written informed consent was obtained from all parents or legal guardians of the children involved in the study. The Research Ethics and Medical Board of the Bambino Gesù Children's Hospital approved the experimental protocol, compliant with the ethical standards outlined in the 1964 Declaration of Helsinki. Experimental trials were performed at the Movement Analysis and Robotic Laboratory (MARLab) at Bambino Gesù Children's Hospital, where patient were recruited.

**Table 1:** **Participants of the two groups involved in the study (HC, Children with Hemiplegia; DC, Children with Diplegia; TDC, Typically Developing Children; MAS, Modified Ashworth Scale; and ROM, Range of Motion)**.

	**ID**	**Age (year)**	**Height (mm)**	**Body mass (kg)**	**Diagnosis**	**Passive ROM (°) right side**	**Passive ROM (°) left side**	**MAS (gastrocnemi)**
HC	#1	5	1195	22	Right hemiplegia	66	69	2
	#2	5	1180	23	Left hemiplegia	67	64	3
	#3	5	1190	26	Right hemiplegia	69	71	1
	#4	5	1220	27	Left hemiplegia	63	59	4
	#5	8	1270	30	Right hemiplegia	62	65	3
	#6	5	1190	23	Right hemiplegia	68	71	1+
	#7	9	1290	32	Right hemiplegia	65	70	2
	#8	9	1300	33	Right hemiplegia	67	69	1+
DC	#9	5	1250	25	Diplegia	64	62	2
	#10	8	1310	34	Diplegia	60	59	4
TDC	#1	6	1280	32	Healthy	70	−	−
	#2	7	1275	31	Healthy	71	−	−
	#3	6	1290	33	Healthy	73	−	−

### Experimental set-up

The pediAnklebot (Krebs et al., 2011; Michmizos et al., 2015) imposes torques and rotations with programmed angular velocities to the ankle joint and, at the same time, measures their values. In this study, the pediAnklebot was used to impose stretch movements, i.e., rotations at different angular velocities to the ankle joint. It is a backdriveable device with low-friction and low mechanical impedance, permitting normal ROM of the foot with respect to the shank in all anatomical planes. Two linear actuators mounted in parallel compose the kinematic design of the mechatronic device. When the actuators push or pull in the same direction a dorsi-plantarflexion torque is produced at the ankle joint, with a maximum torque supplied of 7.21 Nm. Rotations of the ankle in the sagittal plane can be imposed with a ROM from 65° to 135°. The pediAnklebot is actuated by two brushless DC motors (Maxon EC-powermax 22-327739). Two mini-rail linear encoders (MNS9-135 length, Schneeberger), mounted in parallel to the actuators and possessing a resolution of 1 μm, are used to estimate ankle angles in dorsi-plantarflexion. The outputs of two rotary encoders with 40,960 lines are gathered for the servo-amplifier commutation.

**Figure 1:**
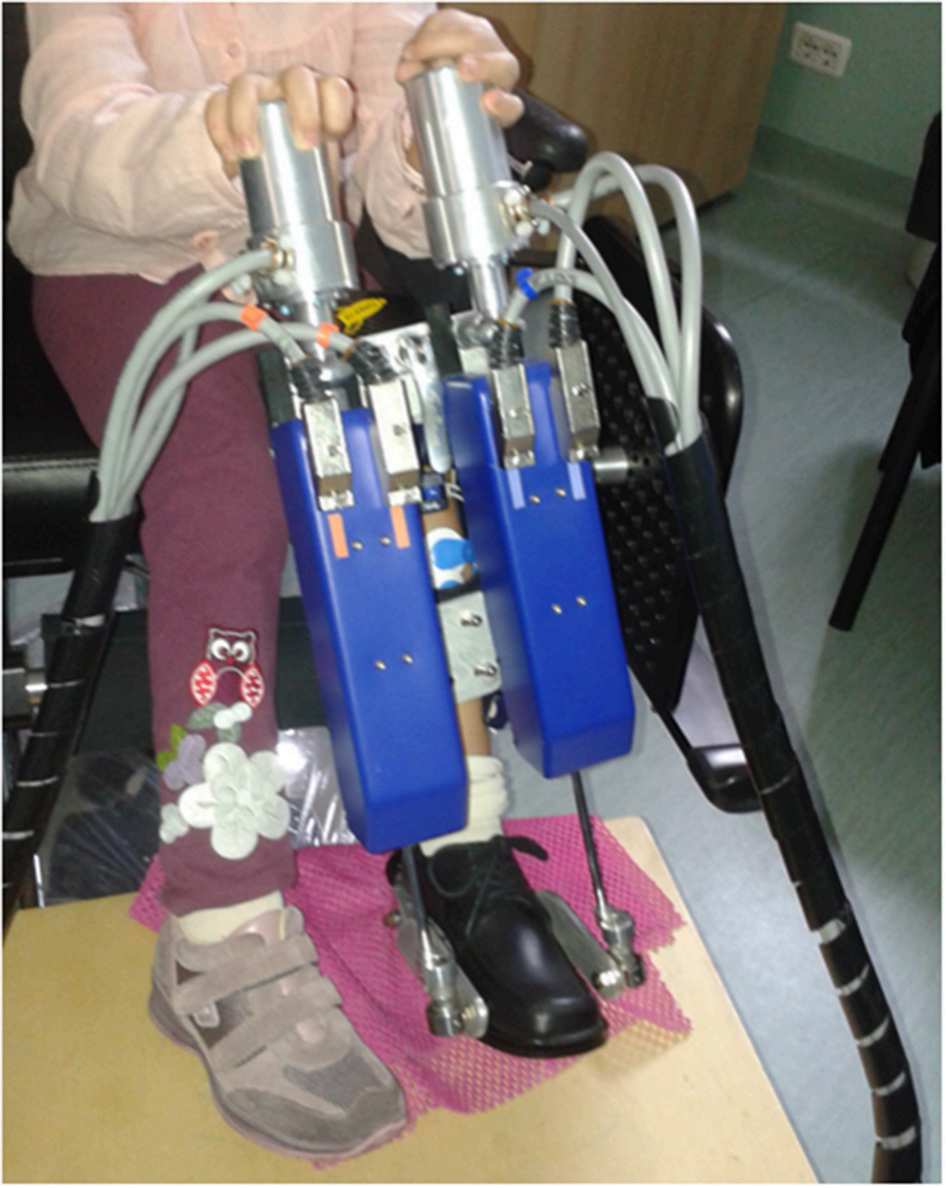
**A children with hemiplegia wearing the PediAnklebot and instrumented with SEMG**.

For measuring muscle activities, passive surface Ag/AgCl circular electrodes (BlueSensor M, Ambu, Ballerup, Denmark) and a wireless SEMG system (Wave, Cometa, Milan, Italy) were applied on tibialis anterior (TA) muscle, involved in dorsiflexion, lateral gastrocnemius (LG), and medial gastrocnemius (MG) muscles, both involved in plantarflexion. Electrode positions and inter-electrode distances were selected in accordance to the SENIAM guidelines (Stegeman and Hermens, 1999). The wireless SEMG system guarantees no time delay in the acquired signal during the experimental acquisition. The entire experimental setup is shown in Figure 1.

### Experimental protocol

**Figure 2:**
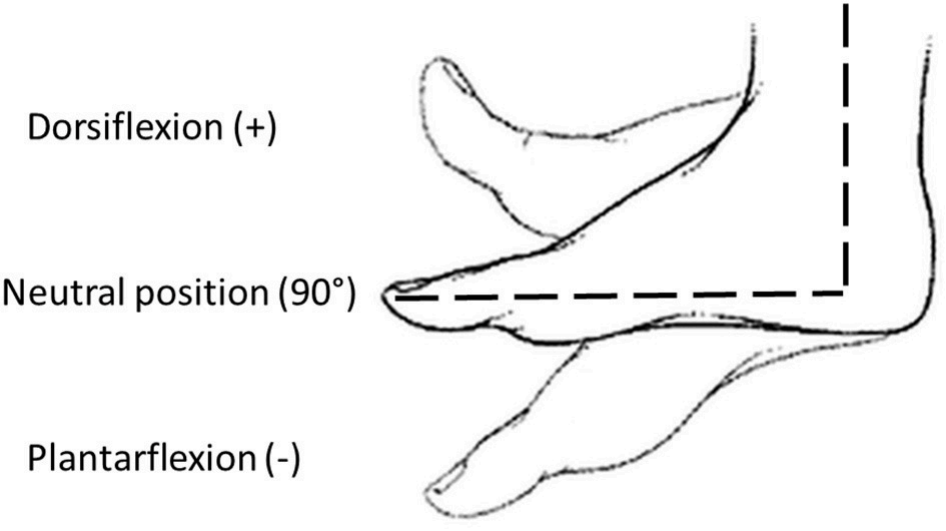
**Definition of joint angles used in the study: neutral position (90°), dorsiflexion (>90°), and plantarflexion (<90°)**.

Before the experimental protocol, spasticity was clinically assessed using the Modified Ashworth Scale (MAS), administered by a skilled physiotherapist. Then, the subject sat comfortably on an adjustable chair and the pediAnklebot was mounted on the lower limb. He/she underwent a familiarization training session, which lasted until participants felt familiar with the equipment. The same physiotherapist placed the ankle in neutral position, i.e., an angle of 90° in sagittal plane between foot and shank (Michmizos et al., 2015), while the foot was resting on a support. The neutral position and the direction of dorsi-plantarflexion are reported in Figure 2. This position was maintained for 10 s to record the baseline muscle activity from the SEMG outputs. Passive ROMs of the ankle joint both in dorsi and plantarflexion were then recorded for each subject. Then, the pediAnklebot imposed passive stretch angles moving the ankle alternatively from the 90% of the dorsiflexion passive ROM to the 90% of the plantarflexion ROM, at five different velocities (50, 100, 150, 200, and 250°/s). For each combination of direction and velocity, the perturbation was applied five times, making a total of 25 stretch movements in dorsiflexion and 25 stretch movements in plantarflexion. To evaluate the accuracy of the pediAnklebot in imposing angular velocities, two preliminary tests were performed. The first test was conducted without the subject's limb linked to the device and the maximum relative error between the imposed and the measured angular velocity was equal to 2%. The second test was performed with the device mounted on a subject and a maximum relative error of 4% was found.

The trial did not start until the participant indicated that he/she was ready to begin, and muscles were relaxed, as showed by the SEMG signals. For each direction, the velocity sequence was randomized and varied among subjects; this variability was introduced to avoid a “muscle accommodation” effect (Vodovnik et al., 1984). To limit the influence of the previous applied stretches on the response to the following, the stretches were separated by a minimum time of 10 s (Mullick et al., 2012). Moreover, an additional variable time (between 0 and 2 s) was added to the minimum time interval to avoid subject anticipation. Therefore, stretches were interspersed with a time interval randomly varying between 10 and 12 s. During each trial, subjects were asked to relax their muscles as much as possible, and, in addition, their attention was captured with the projection of their preferred cartoons, to avoid voluntary interaction with the movement imposed by the robotic device. The cartoon's volume was kept higher than the noise of the actuators, thus children were could not perceive the beginning of the motor's movement. The session lasted approximately 45 min per participant.

SEMG signals of the three above-mentioned muscles, the angular velocity and the angle time histories were acquired. The entire protocol was automatically performed by an *ad-hoc* control algorithm implemented into the pediAnklebot, programmed to impose passive stretches with random velocities, and to synchronize the SEMG with pediAnklebot outputs.

The protocol was performed by the same operator and was repeated for both lower limbs in children with CP, and only for the dominant side in TDC. Dominant side assessment was done by asking to kick a ball (Rossi et al., 2013).

### Data analysis

The data post-processing was performed using Matlab software (MathWorks, 2012b, Natick, MA, USA). According to the literature (Jobin and Levin, 2000; Mullick et al., 2012), raw SEMG signal was firstly filtered with a band-pass (20–500 Hz) 2nd order zero-phase Butterworth filter and, then, a low-pass 30 Hz 6th order zero-phase Butterworth filter was applied to the rectified signal to extract its envelope (Bar-On et al., 2014). The mean value, addressed as baseline, and standard deviation (SD) were evaluated on the rectified SEMG signals that were acquired with the ankle in neutral position in a 100 ms windows before the application of each passive stretch. The stretch reflex onset was defined from the SEMG envelope as the first sustained burst that appeared and remained above the baseline + 3 SDs for at least 15 ms. The joint angles, i.e., the dynamic stretch reflex thresholds (DSRTs), and the angular velocities (ω), corresponding to the stretch reflex onsets, were extracted from data provided by the pediAnklebot. The following linear dependency between DSRT and ω can be assumed as also reported in (Jobin and Levin, 2000; Mullick et al., 2012):

(1) DSRT=TSRT−μω

A linear regression model was applied to the points (DSRT, ω) to compute the value of μ and TSRT. The μ value represents the sensitivity of DSRT to velocity and it is defined as the angular coefficient of the regression line. In particular a positive value of μ indicates a damping response, while a negative value shows an anti-damping velocity-dependent response to muscle stretch. TSRT was defined as the angle at which muscles are actived during quasi-static stretching; thus, TSRT was estimated as the intercept of the regression line with the angle axis. According to Jobin and Levin (Jobin and Levin, 2000), the TSRT values fall into the biomechanical ankle range (from 140° for the plantarflexion to 70° for the dorsiflexion (Evans and Scutter, 2006) in subjects with spasticity, while they are outside the range in typically developing children. Furthermore, TSRT can be related to spasticity severity. In dorsiflexion movements that elicited stretch reflex in MG and LG, values closer 140° are related to a higher pathology severity. Instead, in plantarflexion movements, which elicited stretch reflex in TA, the spasticity severity increases when the TSRT decreases from 140° to 70°.

To evaluate the quality of the linear regression, we used the correlation coefficient (r). The absolute value of r can be interpreted, in agreement to (Dancey and Reidy, 2007), as: (i) no correlation, if |r| ≤ 0.1; (ii) mild/modest correlation, if 0.1 < |r| ≤ 0.3; (iii) moderate correlation, if 0.3 < |r| ≤ 0.6; (iv) strong correlation, if 0.6 < |r| < 1; and, finally, (v) perfect correlation, if |r| = 1.

Before the evaluation of TSRT and μ, SEMG signals were accurately screened to avoid false detections of stretch reflex. Specifically, trials were discarded when following cases occurred (Lynn et al., 2013): (i) simultaneous antagonist muscle activity; (ii) presence of clearly identifiable movement artifacts; and, (iii) continuous muscle activation before or during the imposition of passive stretches. Thus, the linear regression was not performed and the associated metrics was not computed when the number of detected DSRTs was less than 6, in accordance to Ferreira et al. (2010). After data reduction, the median value of DSRT referred to the elicited number of stretch reflexes for plantarflexors and dorsiflexor was calculated, by considering separately the less and more affected sides for HC and DC, and the dominant side for the TDC.

A paired *t*-test on the TSRT values related to MG and LG was performed, considering data gathered from the most affected side of HC and both sides of DC. Test was conducted, with α equal to 0.05, to evaluate if muscles recruited in the same movements show the same behavior in terms of TSRT.

Correlations between TSRT and μ related to the same side were determined using Pearson's coefficient, while correlations between objective indices (TSRT and μ) and MAS were determined using the Spearman's test. A *p* < 0.05 was chosen to indicate statistical significance for all the comparisons. Statistical power of the Spearman's tests was computed using G^*^power software (Faul et al., 2007). Statistical analysis was conducted with SPSS package (IBM-SPSS Inc., Armonk, NY, USA).

## Results

**Figure 3:**
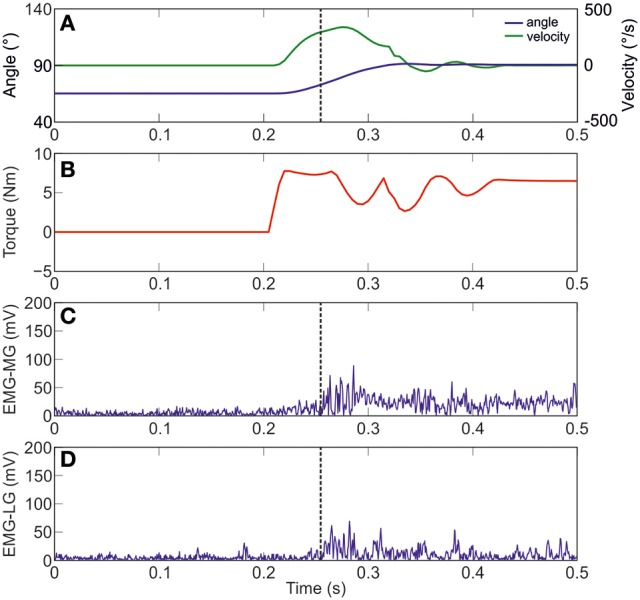
**A representative figure of the imposed angle, angular velocity, torque and relative EMG signals of the Medial and Lateral Gastrocnemius (Patient #10). (A)** imposed angle in blue and angular velocity in green; **(B)** torque; **(C)** raw data of MG; and **(D)** raw data of LG. Dotted black line indicates the stretch reflex onset.

**Figure 4:**
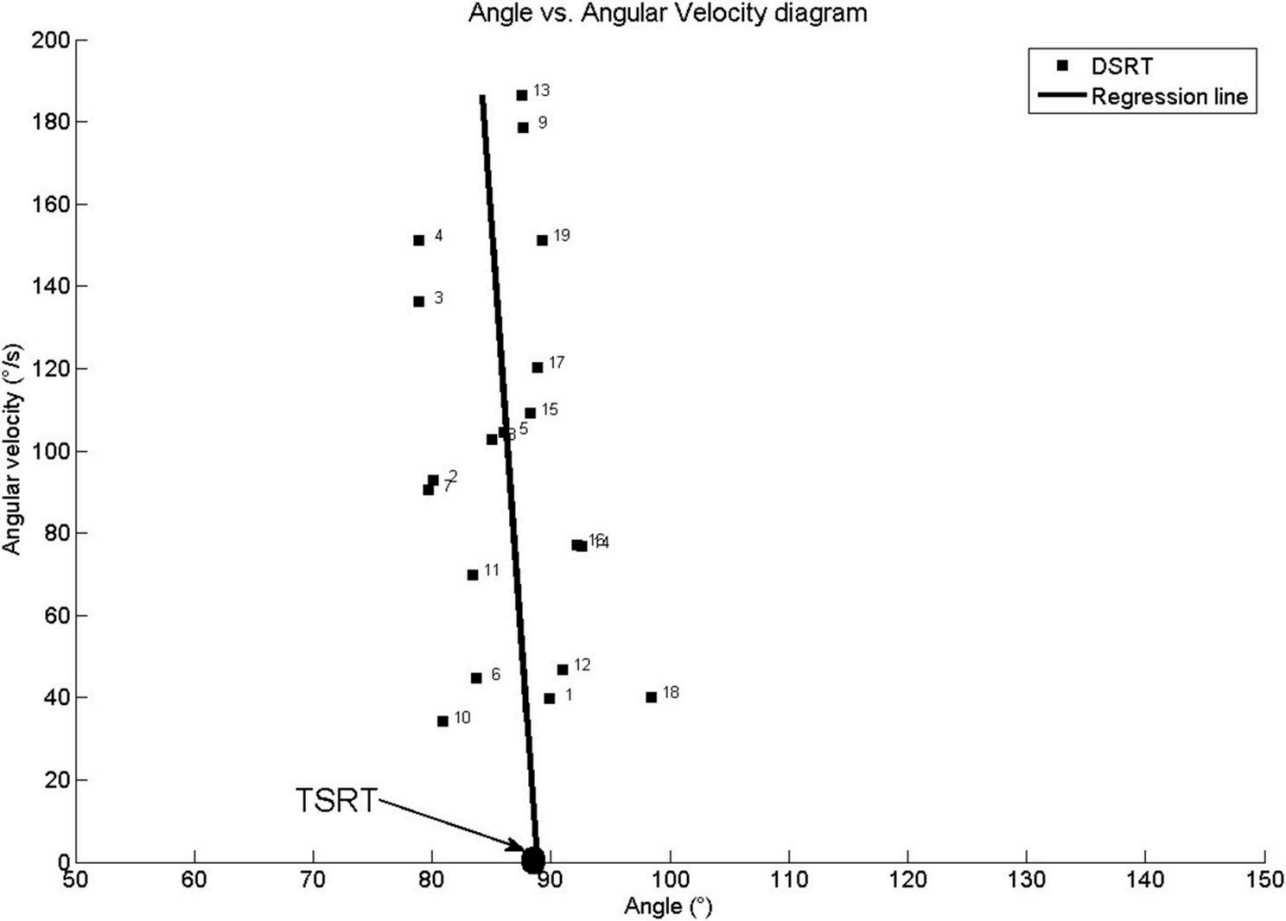
**Patient #1: Angle vs. Angular velocity plot and computed parameters for Medial Gastrocnemius**. TSRT stands for Tonic Stretch Reflex Threshold and DSRT stands for Dynamic Stetch Reflex Threshold.

Figure 3 shows joint angle, angular velocity, torque and relative EMG signals of the plantarflexor responses for a representative patient. A paradigmatic relationship between angular velocity and joint angle, along with the regression line and the related parameters is reported in Figure 4. The values of Dynamic Stretch Reflex Threshold DSRT, Tonic Stretch Reflex Threshold TSRT, the sensitivity to stretch reflex μ and the coefficient of correlation r relative to the three examined muscles for all subjects are reported in Table 2.

**Table 2:** **Dynamic Stretch Reflex Threshold (DSRT), Tonic Stretch Reflex Threshold (TSRT) and slope (μ) of the line interpolating velocities with DSRT values for all subjects in each group relative to Medial Gastrocnemius (MG), Lateral Gastrocnemius (LG), and Tibialis Anterior (TA)**.

**Participants**		**MG**				**LG**				**TA**			
		**N° DSRT**	**TSRT (°)**	**μ (s)**	**r**	**N° DSRT**	**TSRT (°)**	**μ (s)**	**r**	**N° DSRT**	**TSRT (°)**	**μ (s)**	**r**
HC	#1 MA	20	84.2	0.062	−0.52	20	90.4	0.034	−0.31	4	ND	ND	ND
	#2 MA	15	99.2	0.008	−0.33	16	97.6	0.016	−0.30	5	ND	ND	ND
	#3 MA	12	77.3	0.123	−0.82	17	90.3	0.059	−0.36	8	71.1	0.026	−0.68
	#4 MA	14	94.5	0.082	−0.32	14	81.3	0.094	−0.33	4	ND	ND	ND
	#5 MA	16	101.0	−0.050^*^	0.32	6	70.1	0.105	−0.75	5	ND	ND	ND
	#6 MA	10	96.3	0.057	−0.34	15	103.5	0.051	−0.44	20	61.7^#^	0.051	−0.92
	#7 MA	14	126.2	0.014	−0.20	14	112.9	0.107	−0.47	19	91.0	0.026	−0.56
	#8 MA	13	93.0	0.046	−0.49	9	70.2	0.200	−0.84	13	80.0	0.035	−0.71
DC	#9 MA	13	88.1	0.109	−0.45	13	55.6^#^	0.252	−0.58	0	ND	ND	ND
	#10 MA	15	97.2	0.027	−0.32	14	95.1	0.029	−0.30	1	ND	ND	ND
HC	#1 LA	11	105.3	−0.048^*^	−0.43	4	ND	ND	ND	6	77.9	0.012	−0.52
	#2 LA	8	91.8	0.022	−0.48	9	104.4	−0.026^*^	−0.27	5	ND	ND	ND
	#3 LA	3	ND	ND	ND	5	ND	ND	ND	10	74.7	0.022	−0.37
	#4 LA	12	94.1	0.090	−0.39	12	89	0.560	−0.32	9	114.0	−0.142^*^	0.56
	#5 LA	3	ND	ND	ND	3	ND	ND	ND	5	ND	ND	ND
	#6 LA	3	ND	ND	ND	8	126.5	−0.062^*^	0.30	9	100.0	−0.063^*^	0.59
	#7 LA	3	ND	ND	ND	5	ND	ND	ND	5	ND	ND	ND
	#8 LA	10	77.3	0.141	−0.71	10	114.1	−0.155^*^	0.41	5	ND	ND	ND
DC	#9 LA	10	82.7	0.162	−0.82	10	87.5	0.135	−0.57	11	84.5	−0.380^*^	0.45
	#10 LA	10	90.8	0.089	−0.61	10	99.6	0.042	−0.30	1	ND	ND	ND
	#1	9	49.1^#^	0.183	−0.51	9	65.2^#^	0.009	−0.36	11	53.1^#^	0.021	−0.71
TDC	#2	9	56.8^#^	0.117	−0.62	8	69.6^#^	0.114	−0.35	11	59.4^#^	0.025	−0.51
	#3	8	42.0^#^	0.242	−0.60	10	67.7^#^	0.106	−0.52	1	ND	ND	ND
r range		No		Modest		Strong		Perfect					

*ND stands for “not−definable*.”

* *represents the case in which the angular coefficient of regression line is negative*.

# *represents the case in which TSRT value is outside the biomechanical ROM. MA indicates the most affected side and LA the less affected one*.

### Dynamic stretch reflex threshold (DSRT)

As regards the most affected side of children with CP, passive stretches were able to elicit the stretch reflex of the muscles involved in the plantarflexion, i.e., MG and LG, as demonstrated by the number of DSRT, which resulted never lower than six and its median was equal to 14. Conversely, for the muscle involved in the dorsiflexion, TA, the number of DSRT was greater than 6 only in four cases and its median was equal to 16. Furthermore, the number of elicited DSRT was lower in the less affected side than in the most affected one. In particular the number of DSRT was greater than 6 in seven (median equal to 10) and four (median equal to 9) cases for plantarflexor and dorsiflexor muscles, respectively. In typically developing children, the stretch reflex was elicited except for TA and for one subject. The median was equal to 9 for MG and LG and 11 for TA.

### Tonic stretch reflex threshold (TSRT)

In the most affected side of children with hemiplegia and diplegia, all computed TSRTs were into the biomechanical ROM, with the exception of those of patients #6 and #9 for the TA and LG, respectively. More specifically, the mean and the SD of TSRT values were 95.7° (12.9°) and 86.7° (17.4°) respectively for MG and LG, and 75.9° (12.5°) for TA. In the less affected side, no patient showed a TSRT value outside the biomechanical ROM. The mean and the SD of TSRT values were 90.3° (9.7°) and 103.5° (14.9°) for MG and LG, respectively, and 90.2° (16.5°) for TA. In TDC group, TSRT values were always outside the biomechanical range both in dorsiflexion and in plantarflexion; the mean and the SD of TSRT values were 49.3°(7.4°) and 67.5°(2.2°) respectively for MG and LG, and 56.2°(4.4°) for TA.

### Sensitivity to stretch reflex (μ)

Positive values of sensitivity to stretch reflex μ were found in 82% of the computed linear regressions. Negative values of μ occurred in eight cases, one for the most affected side and seven for the less affected one. Specifically, for plantarflexor muscles of the most affected side, the mean value of μ was 0.078 and only subject #5 showed an inverse sensitivity to stretch reflex (μ = −0.050) related to MG. For dorsiflexor muscle, the mean μ value was equal to 0.034. In the less affected side, 67% (mean μ = 0.155) and 40% (mean μ = 0.017) of positive sensitivity values were found for plantarflexor and dorsiflexor muscles, respectively. In TDC, no subject showed negative values of μ. In particular, we found mean values of μ equal to 0.128 both in MG and LG, and 0.023 in TA.

### Correlation coefficient r

Considering the plantarflexor muscles of the most affected side, the r values lied in the range of moderate to strong correlation, with one exception of no correlation (*r* = −0.20) for MG in patient #7. The observed mean value for r was equal to −0.44, ignoring the positive value obtained for MG in patient #5. As concerns dorsiflexor muscle, moderate to strong correlations were observed for all patients with a mean value equal to −0.72. In the less affected side, moderate to strong negative correlations were found both for plantarflexor and dorsiflexor muscles, with mean values equal to −0.52 and −0.44, respectively. Moreover, seven occurrences of positive, from modest to moderate, correlations were observed, four in MG and LG, and three in TA.

In TDC, all coefficients were in the range from moderate to strong correlation for all muscles and no positive correlation was found.

### Correlations between MAS and TSRT, MAS, and μ, μ and TSRT

We did not administer the MAS scale for TA, since the spasticity phenomenon mostly affects the plantarflexors (Mazlan et al., 2012).

Spearman's and Pearson's coefficients (r) and relative *p-*values are showed in Table 3. Power of the test resulted equal to 70% with a medium effect size (0.5) (Cohen, 1988). All tests should be interpreted as exploratory data analysis. Thus, such a power level can be considered acceptable for the present study.

**Table 3:** **Spearman's and Pearson's coefficients r related to the performed correlations and relative (*p*-value) related to Medial Gastrocnemius (MG), Lateral Gastrocnemius (LG), and Tibialis Anterior (TA). Empty cells indicate not performed correlation**.

	**Muscles**	**Parameters**	**Clinical score**	**MG**		**LG**		**TA**	
			**MAS**	**TSRT**	**μ**	**TSRT**	**μ**	**TSRT**	**μ**
Clinical score		MAS		−0.49 (0.15)	−0.41 (0.23)	−0.06 (0.88)	−0.13 (0.72)		
Most affected side	MG	TSRT	−0.49 (0.15)		0.70 (0.04)^*^	0.47 (0.17)	0.01 (0.98)	0.76 (0.24)	0.47 (0.93)
		μ	−0.41 (0.23)	0.70 (0.04)^*^		0.18 (0.62)	0.24 (0.50)	0.51 (0.50)	0.31 (0.55)
	LG	TSRT	−0.06 (0.88)	0.47 (0.17)	0.18 (0.62)		0.74 (0.01)^*^	0.45 (0.55)	−0.49 (0.32)
		μ	−0.13 (0.72)	0.01 (0.98)	0.24 (0.50)	0.74 (0.01)^*^		−0.18 (0.82)	0.80 (0.06)
	TA	TSRT		0.76 (0.24)	0.51 (0.50)	0.45 (0.55)	−0.18 (0.82)		0.74 (0.26)
		μ		0.47 (0.93)	0.31 (0.55)	−0.49 (0.32)	0.80 (0.06)	0.74 (0.26)	
Less affected side	MG	TSRT			0.90 (0.01)^*^	−0.53 (0.27)	−0.56 (0.33)	−0.06 (0.96)	−0.99 (0.07)
		μ		0.90 (0.01)^*^		−0.17 (0.75)	0.07 (0.91)	−0.24 (0.84)	−0.95 (0.19)
	LG	TSRT		−0.53 (0.27)	−0.17 (0.75)		0.86 (0.03)^*^	0.22 (0.78)	−0.37 (0.63)
		μ		−0.56 (0.33)	0.07 (0.91)	0.86 (0.03)^*^		−0.04 (0.97)	−0.64 (0.56)
	TA	TSRT		−0.06 (0.96)	−0.24 (0.84)	0.22 (0.78)	−0.04 (0.97)		0.17 (0.78)
		μ		−0.99 (0.07)	−0.95 (0.19)	−0.37 (0.63)	−0.64 (0.56)	0.17 (0.78)	

*Spearman's test was used for correlation between MAS and TSRT and μ, while Pearson's test for correlation between TSRT and μ*.

* *marks significant correlations*.

MAS values did not show any correlation with the objective indices computed for the most affected side. By comparing TSRT and μ values of different muscles, no statistical correlation was found for both lower limb sides. Conversely, a positive strong correlation was found both for MG and LG between TSRT and μ computed for the same muscle on both most affected and less affected sides. A similar effect was not observed in TA.

## Discussion

The present study explored the feasibility of using a robotic device for the assessment of spasticity at the ankle joint in children with CP, through the TRST approach. The obtained metrics was compared to the MAS clinical scale.

### Is the TSRT approach feasible for the spasticity assessment in medial gastrocnemius, lateral gastrocnemius, and tibialis anterior?

The proposed experimental protocol has the potential to elicit a high number of stretch reflexes in medial and lateral gastrocnemius muscles. Therefore, it confirms the results obtained by Blanchette et al. (2015) in adult post-stroke patients on the same joint. The here adopted approach is sensitive to pathology severity, eliciting a higher DSRT number in the most affected side than the less affected one.

Conversely, the assessment of spasticity via TSRT approach for the tibialis anterior cannot be performed due to the low number of gathered DSRT, i.e., lower than the generally accepted threshold value of 6 (Ferreira et al., 2010). The reduced capability in eliciting a high number of stretch reflexes on the tibialis anterior can be ascribed to the absence of the hyperexcitability phenomenon, as it is generally understood that, in children with CP, spasticity mainly affects plantarflexor muscles (Engsberg et al., 2007). The observed low values of DSRT for the TA in the present experimental study cannot be compared with those of previous studies, due to the novelty in both the targeted muscle and the population.

Focusing on TSRT, the entire set of computed values for the three examined muscles of the most affected side lied into the biomechanical ROM, with the only exception of patient #9 in LG and patient #6 in TA. This outcome demonstrates that spasticity is related to a hypertonicity phenomenon, causing a decrease in the range of central regulation of tonic stretch reflex, and the inability to prevent activation of muscle within biomechanical range (Mullick et al., 2012). The ability to regulate tonic stretch reflex threshold in a range outside the biomechanical ankle ROM is crucial for the movement control (Mullick et al., 2012). Deficit in TSRT regulation is considered a leading cause of instability, weakness, and impairments in interjoint coordination (Musampa et al., 2007). Moreover, the regulation of the stretch reflex permits to convert the reflex resistance in the force that assists the motion (Feldman and Latash, 2005). Although no statistical difference was found between TSRT in MG and LG (*p* = 0.12), we observe that in some patients (HC#3, HC#5, HC#8) values of TSRT were different between MG and LG. This finding suggests that the hyperexcitability hides *de-facto* a complex interaction between central and peripheral contributions to spasticity. Thus, muscles recruited in the same movement can differently contribute depending on the pathology severity.

The observed differences in TSRT values among patients with the same MAS score might be due to the subject-specific response to the externally imposed joint rotations, as well as by the intrinsic inability of the MAS to discriminate neural from non-neural components. The computed μ values confirm the outcomes described by Blanchette et al. (2015), who demonstrated a lower sensitivity to the stretch velocity of the ankle plantarflexors than the elbow muscles. This outcome could be ascribed to the higher passive stiffness of the ankle joint with respect to the elbow one (Given et al., 1995). The negative values of μ prove an atypical opposite dependency of DSRT to the velocity. The lower the velocity of the imposed rotations the earlier occurs the stretch response. This effect might be due to the common occurrence of muscle shortening reactions in patients with neuromotor diseases (Andrews et al., 1972). Moreover, a low sensitivity to stretch reflex suggests a strong presynaptic inhibition of primary fiber of type Ia afferent discharges (Mullick et al., 2012).

The computed correlation coefficients r for plantarflexor (MG and LG) and dorsiflexor (TA) muscles were lower than those obtained by using the same methodology at the elbow joint (Jobin and Levin, 2000; Mullick et al., 2012), or at the ankle joint for adult post-stroke patients (Blanchette et al., 2015). We speculate that the previously indicated outcomes could be influenced by: (i) the lower ROM value for the ankle compared to the elbow one; (ii) the targeted children population; and (iii) the level of the pathology, which is mild or slight in the recruited patients. The low values of correlation coefficient could also be ascribed to the lower cooperation of children with respect to adults (Ferreira et al., 2013), leading to a higher number of discarded trials for the presence of voluntary movements. Similarly, the observed differences in the r values related to the MG and LG in the same patient could be due to the different number of DSRT values used for the computation of the regression line, as also reported by Ferreira et al. (2010).

In typically developing children, the computed TSRT values confirm that healthy children are able to control stretch responses outside the biomechanical range, providing full extension of the ankle ROM in the sagittal plane. The DSRT number different from zero confirms the findings of Pisano et al. (2000), who argued the possibility to evoke involuntary muscle responses also in healthy individuals.

The positive correlation between TSRT and μ computed for MG, LG of both sides, confirmed that higher TSRT values imply a higher slope of the regression line, as reported by Jobin and Levin (2000). Conversely, in correspondence of low TSRT values we found low μ values. This finding suggests that a more stable response to the stretch reflex is reached. The absence of significant correlations between TSRT and μ in TA suggests that this relationship only exists in muscles affected by spasticity.

As a general conclusion, the present study demonstrates the feasibility of using a robotic device for the evaluation of spasticity in plantarflexors muscles at the ankle joint in children with CP, through the TRST approach. Conversely, the same approach appears to be not consistent for assessing spasticity of the dorsiflexor. Finally, it should be noted that a potential limitation of this approach could be in mapping linear velocity of muscle stretches through angular velocity of the joint. Even though the estimation of the linear velocity of muscle stretches via a neuro musculoskeletal simulation for assessing spasticity was recently proposed (van der Krogt et al., 2016), the large amount of scientific researches based on TSRT approach suggests that the use of angular velocity still remains the most widespread methodology for spasticity assessing, due to its easier applicability in clinical routine.

### Is there any correlation between objective measures and clinical scales?

No correlation between MAS score, TSRT, and μ values was observed. This result confirms the findings reported by Mullick (Mullick et al., 2012) and Jobin (Jobin and Levin, 2000) about the weak or no-correlation between TSRT and MAS score at the elbow joint, in adults post stroke and children with cerebral palsy, respectively. The absence of correlation might be due to different physiological parameters assessed via the two indices. MAS scale is supposed to evaluate the supra-threshold resistance to passive movement (Mullick et al., 2012), while TSRT measures the limitation in the range of central regulation of the tonic stretch reflex threshold (Blanchette et al., 2015), neglecting the supra-threshold resistance (Mullick et al., 2012). Moreover, MAS depends on the viscoelastic properties of the muscles. Thus, the resistance to external movements could be perceived when no-contractile components of the muscles change (Jobin and Levin, 2000). Conversely, variations in the mechanical properties of the muscles are not identified by the TSRT, as they do not cause EMG activity (Jobin and Levin, 2000). Considering the different methodological approaches for the evaluation of MAS and TSRT, the absence of correlation between the two indices is not surprising. Consequently, we suggest to use TSRT outputs and MAS score together, enhancing the information about the physiological mechanisms due to deficits in muscle tone.

The observed differences in TSRT values among patients with the same MAS score might be justified by the subject-specific response to the externally imposed joint rotations and the intrinsic inability of the MAS to discriminate neural from non-neural components. The correlation between neural and non-neural components could be evaluated, in future investigations, by examining the biomechanics of the gait. It is generally understood, in fact, that these two components belongs to two different logical levels. Specifically, non-neural components represent a system of constrains and peripheral resources, while neural components utilize these peripheral resources to balance the movement dynamics (Wood et al., 2005). Gait analyses might be added to TSRT and MAS evaluations, for a more exhaustive insight in the spasticity phenomenon.

## Conclusion

In this paper, we demonstrated the feasibility of applying the TSRT approach for the assessment of spasticity of the ankle plantarflexor muscles in children with Cerebral Palsy, by means of a mechatronic device. No correlation was found between the proposed objective measures and the clinical scores based on Modified Ashworth Scale.

Future investigations would integrate the Tonic Stretch Reflex Threshold approach with functional observations, such as gait analysis, permitting a more deeply understanding of the relationship between motor performance and muscles excitability.

## Ethical standards

All procedures performed in studies involving human participants were in accordance with the ethical standards of the institutional research committee. The study complied with the principles of the Declaration of Helsinki and the protocol was approved by the Research Ethics Board of the “Bambino Gesù” Children's Hospital. The Ethics Committee of “Bambino Gesù” Children's Hospital approved this experimental study n.548/2012 with approval number 597/12 on 10th October 2012. Informed consent, in written form, was obtained from all parents or legal guardians of the children involved in the study.

## Author contributions

All authors contributed to the experimental design. MG and JT performed the literature survey. MG, FF, and MP performed patients' recruitment and data collection. MG, JT, and SR analyzed the data. MG, JT, SR, PC, EP, and MP wrote the manuscript. PC, EC, and MP cooperated in funding research and research management, supervising the research project. All the authors reviewed the manuscript and approved the final version.

## Author note

Paolo Cappa passed away on August 26th 2016. This article was seen and approved by him in early August 2016.

### Conflict of interest statement

The authors declare that the research was conducted in the absence of any commercial or financial relationships that could be construed as a potential conflict of interest.

## Abbreviations

μ: sensitivity to the stretch reflex
ω: angular velocity
CP: Cerebral Palsy
DC: Children with Diplegia
DSRT: Dynamic Stretch Reflex Threshold
HC: Children with Hemiplegia
LG: Lateral Gastrocnemius
MAS: Modified Ashworth Scale
MG: Medial Gastrocnemius
r: correlation coefficient
ROM: Range Of Motion
SD: Standard Deviation
SEMG: Standard Deviation
TA: Tibialis Anterior
TDC: Typically developing children
TSRT: Tonic Stretch Reflex Threshold.
